# Enhanced efficacy of 0.05% cyclosporine a combined with absorbable punctal plugs in Sjögren’s syndrome-associated dry eye: a paired-eye clinical trial

**DOI:** 10.3389/fmed.2025.1674647

**Published:** 2026-01-06

**Authors:** Yi Dong, Jin-fu Cao, Ze-cheng Li, Xin-yu Yang, Luxia Chen

**Affiliations:** 1 Tianjin Eye Hospita, Tianjin Key Lab of Ophthalmology and Visual Science, Nankai University Affiliated Eye Hospital, Tianjin, China; 2 Clinical College of Ophthalmology, Tianjin Medical University, Tianjin, China

**Keywords:** Sjögren’s syndrome, dry eye, cyclosporine a, absorbable punctal plugs, paired-eye study

## Abstract

**Introduction:**

To compare the efficacy of combination therapy using 0.05% cyclosporine A (CsA) and absorbable punctal plugs with that of CsA monotherapy in patients with Sjögren’s syndrome-associated dry eye (SS-DE) using a paired-eye randomized controlled design.

**Methods:**

This prospective, randomized, observer-masked, paired-eye clinical trial included 21 patients (42 eyes) with SS-DE. One eye of each patient received 0.05% CsA plus an absorbable lower punctal plug, whereas the contralateral eye received CsA monotherapy. Clinical assessments, including the Ocular Surface Disease Index (OSDI), visual analog scale (VAS), noninvasive tear breakup time (NIBUT), tear meniscus height (TMH), and corneal dendritic cell (DC) density (measured via *in vivo* confocal microscopy), were conducted at baseline and after 3 months. Intra-and intergroup comparisons were performed using paired t-tests. Pearson’s correlation and linear regression analyses were used to evaluate the association between ΔVAS and ΔOSDI.

**Results:**

After 3 months, both groups showed significant improvements across all parameters (*p* < 0.01). The combined treatment group demonstrated significantly greater improvements in NIBUT (*p* < 0.01), TMH (*p* < 0.05), DC density (*p* < 0.01), and VAS scores (*p* < 0.01) than the CsA monotherapy group. A significant positive correlation between ΔVAS and ΔOSDI was found in the combined treatment group (r = 0.528, *p* = 0.014), but not in the CsA monotherapy group (r = −0.172, *p* = 0.456).

**Conclusion:**

Combination therapy with 0.05% CsA and absorbable punctal plugs yields superior improvements in both objective clinical signs and subjective symptoms compared to CsA monotherapy in SS-DE. This approach may enhance drug efficacy and patient comfort, offering an optimal therapeutic strategy for autoimmune-related dry eye.

**Clinical trial registration:**

[ClinicalTrials.gov] Identifier: NCT07171710.

## 1 Introduction

Sjögren’s syndrome (SS) is a chronic systemic autoimmune disease characterized by lymphocytic infiltration of exocrine glands, including the lacrimal and salivary glands, leading to xerophthalmia and xerostomia (1). Dry eye is a prominent and disabling ocular manifestation of SS. Compared with non-SS dry eye, SS-associated dry eye (SS-DE) presents with more severe symptoms, heightened ocular surface inflammation, and an increased risk of corneal complications (2). Ocular surface tissues in patients with SS-DE often exhibit elevated levels of T lymphocyte infiltration and inflammatory cytokines, which contribute to disease progression and treatment resistance (2, 3).

Topical cyclosporine A (CsA) is a widely used immunomodulatory agent for SS-DE (4). Although effective, its therapeutic onset is slow, and efficacy may be limited by rapid tear drainage through the nasolacrimal system. Absorbable punctal plugs provide a noninvasive, biocompatible method of temporary punctal occlusion to retain natural tears, extend drug contact time, and improve treatment efficacy (5). Unlike permanent plugs, absorbable plugs self-resorb without the need for removal and are associated with minimal risk of adverse effects (6, 7).

This study aimed to compare the efficacy of combination therapy using 0.05% CsA and absorbable punctal plugs with that of CsA monotherapy in patients with SS-DE.

## 2 Materials and methods

### 2.1 Participants and enrollment

This prospective, randomized, controlled, observer-masked, paired-eye clinical trial was conducted at Tianjin Eye Hospital, a tertiary class A hospital in China. The study adhered to the tenets of the Declaration of Helsinki and was approved by the Medical Ethics Committees of Tianjin Eye Hospital (Approval Number: KY-2023045). This study was registered at ClinicalTrials.gov (Identifier: NCT07171710). Written informed consent was obtained from all participants before enrollment.

Eligible participants were adults aged ≥ 18 years with a confirmed diagnosis of SS. All patients were diagnosed by rheumatologists following the 2016 Sjögren’s International Collaborative Clinical Alliance (SICCA) diagnostic criteria (8). Dry eye diagnosis required both subjective and objective criteria. Subjective criteria included one or more ocular surface symptoms, such as dryness, foreign body sensation, burning, fatigue, discomfort, redness, or fluctuating vision, with an Ocular Surface Disease Index (OSDI) score ≥ 13. Objective criteria included a noninvasive tear break-up time (NIBUT) ≤ 10 s and Schirmer I test result ≤ 5 mm/5 min (9).

The exclusion criteria were as follows:

- Structural eyelid abnormalities (e.g., cicatricial changes, entropion, trichiasis)
- Any signs of active intraocular inflammation or anterior segment abnormalities
- History or diagnosis of glaucoma
- History of ocular surgery or punctal closure
- Use of topical ophthalmic medications other than artificial tears, 0.1% fluorometholone, or 0.05% CsA within the past month
- Use of systemic or topical antimicrobial or anti-inflammatory agents within 90 days before the study
- Contact lens wear during the study period
- Active corneal infections
- Corneal diseases, including marginal ulcers, scarring, opacity, bullous keratopathy, conjunctivochalasis, symblepharon, or ocular surface neoplasia
- Pregnancy or lactation
- Changes in systemic immunosuppressive therapy within 90 days before enrollment

Participants meeting all eligibility criteria were enrolled and randomized to receive the combined treatment (0.05% CsA plus an absorbable punctal plug) in one eye and 0.05% CsA monotherapy in the contralateral eye. Eye allocation (left or right) was determined using block randomization with a fixed block size of two, implemented via an online randomization tool.^1^ The randomization sequence was generated in advance by an independent biostatistician who was not involved in patient care or data analysis.

Treatment allocation was concealed using sequentially numbered, opaque, sealed envelopes, prepared by the same biostatistician. At the time of enrollment, the study coordinator opened the next envelope sequentially to determine which eye would receive the combined treatment, while the contralateral eye received CsA monotherapy.

To maintain masking, the ophthalmologists responsible for outcome assessments (including ocular surface staining, tear film measurements, and *in vivo* confocal microscopy) were blinded to treatment allocation. Participants were informed that both eyes would receive active treatment and were not informed of the specific allocation. Investigators who performed punctal plug insertion and administered CsA were not involved in any of the clinical evaluations.

Thirty patients with SS-DE were screened for enrollment. All participants were under regular follow-up by board-certified rheumatologists at the time of enrollment, and their systemic immunosuppressive regimens remained stable throughout the 3-month study period. All participants were under regular follow-up by board-certified rheumatologists at the time of enrollment, and their systemic immunosuppressive regimens remained stable throughout the study period. Sample size was calculated using G*Power 3.1 software (version 3.1.9.7) (10). A two-tailed paired-samples t-test was applied with an effect size (dz) of 0.7, *α* = 0.05, power = 0.80, and an assumed correlation coefficient of 0.5 between paired eyes. The estimated minimum required sample size was 19 patients. Considering potential dropouts, we planned to enroll 30 participants.

#### 2.1.1 Treatment

In this paired-eye study, both eyes of each participant received 0.05% CsA ophthalmic solution (Xingqi Pharmaceutical Co., Shenyang, China) three times daily (morning, afternoon, and evening). In the monotherapy group, one eye was treated with CsA alone throughout the study period. No additional topical medications, lubricants, or anti-inflammatory agents were administered. Patients were instructed on standardized drop instillation techniques, and treatment compliance was monitored via patient diaries and follow-up interviews.

All participants were instructed on standardized drop instillation techniques, and were provided with written dosing schedules to reinforce correct usage. To ensure adherence, patients were required to maintain daily treatment diaries, which were reviewed at scheduled follow-up visits. Additionally, weekly telephone reminders were conducted to reinforce compliance, address any difficulties, and encourage continued participation. These measures were implemented to minimize variations in patient behavior and ensure consistent drug administration across all study participants.

In the combined treatment group, the contralateral eye received 0.05% CsA at the same dose and frequency, along with a single absorbable punctal plug (Dissolvable Visiplug; Lacrimedics Inc., Eastsound, WA, USA) inserted into the lower punctum. Plugs were inserted according to the manufacturer’s instructions. The lower punctal area was gently dried before insertion, and the plug was placed into the vertical canaliculus using sterile forceps, without the need for anesthesia. Proper positioning was confirmed by slit-lamp biomicroscopy immediately after insertion. Plug placement was performed on the same day as treatment initiation, and patients were advised to avoid rubbing the periocular area to prevent dislodgment. To maintain subject masking, a similar sham procedure was applied to the monotherapy eye without actual plug insertion.

### 2.2 Examination

All clinical assessments were conducted at baseline and after 3 months of treatment under standardized environmental conditions (temperature- and humidity-controlled). To ensure consistency and minimize reflex tearing and testing bias, examinations were performed in the following fixed order for each eye:

#### 2.2.1 OSDI

Subjective dry eye symptoms were evaluated using a validated 12-item OSDI questionnaire. The total score ranged from 0 to 100, with higher scores indicating more severe symptoms.

#### 2.2.2 Visual analog scale (VAS)

Participants rated their overall ocular discomfort on a VAS, where 0 indicated no discomfort and 10 cm indicated maximal discomfort. The VAS score served as a quantitative subjective measure.

#### 2.2.3 Tear Meniscus height (TMH)

Lower TMH was assessed using a Keratograph 5 M system (Oculus, Wetzlar, Germany). Measurements were taken at the central lower lid margin and expressed in millimeters (mm). Three values were obtained and averaged for each eye.

#### 2.2.4 NIBUT

NIBUT was also measured using a Keratograph 5 M. Participants were instructed to blink naturally and keep their eyes open. The time to first tear film disruption was automatically recorded. Three consecutive measurements were taken, and the mean value was used for analysis.

#### 2.2.5 Corneal dendritic cell density (DC density)

*In vivo* confocal microscopy was performed using a Heidelberg Retina Tomograph III with a Rostock Cornea Module (Heidelberg Engineering GmbH, Germany). Central corneal scans of the sub-basal nerve plexus were acquired. Dendritic cells were identified based on their hyperreflective cell bodies and dendritic processes. Five high-quality images per eye were analyzed by two independent, masked observers, and the average cell density (cells/mm^2^) was calculated.

### 2.3 Statistical analysis

All statistical analyses were performed using SPSS version 25.0 (IBM Corp., Armonk, NY, USA). The Shapiro–Wilk test was used to assess the normality of data distribution. For normally distributed variables, paired eyes were compared using the paired t-test. The Wilcoxon signed-rank test was used for non-normally distributed variables. Data are presented as mean ± standard deviation for normally distributed variables, and as median with interquartile range for non-normally distributed variables. The correlation between changes in VAS and OSDI scores in each group was analyzed using Pearson’s correlation. Linear regression analysis was also performed to further illustrate this relationship, with the coefficient of determination (R (2)) and corresponding *p*-values reported. A two-sided p-value < 0.05 was considered statistically significant.

## 3 Results

### 3.1 Participant enrollment and baseline characteristics

**Figure 1:**
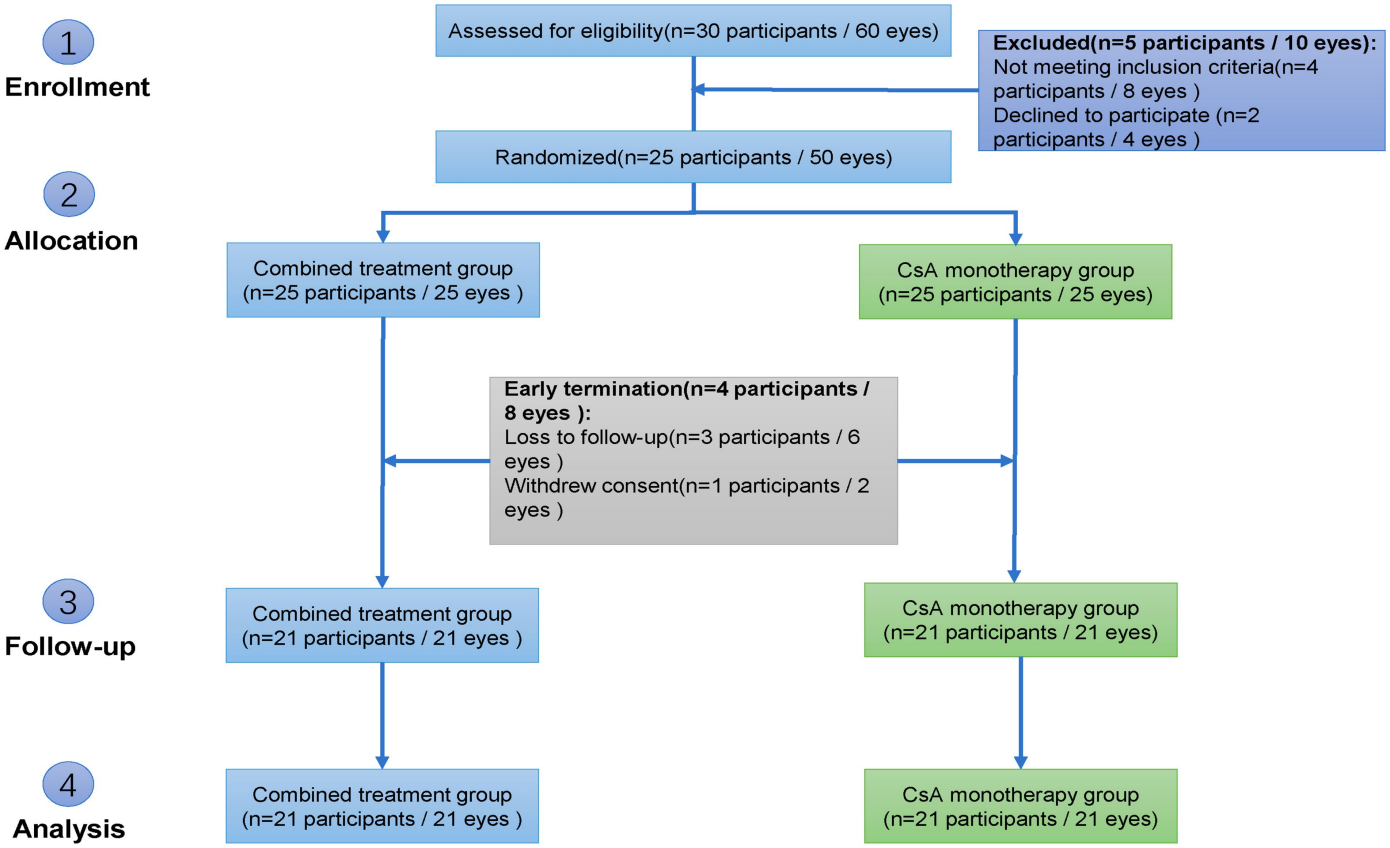
Flowchart of participant enrollment.

As illustrated in Figure 1, 30 participants (60 eyes) were screened for eligibility. Five participants (10 eyes) were excluded: four did not meet the inclusion criteria, and one refused to participate. The remaining 25 participants (50 eyes) were enrolled and randomized using a block randomization method with a paired-eye design. For each participant, one eye was assigned to the combined treatment group (CsA plus absorbable punctal plug) and the contralateral eye was assigned to the CsA monotherapy group.

During follow-up, four participants (eight eyes) discontinued the study: three were lost to follow-up and one withdrew consent. A total of 21 participants (42 eyes) completed the study and were included in the final analysis.

The mean age of the participants was 43.33 ± 15.33 years, with 8 men (38.1%) and 13 women (61.9%). The mean OSDI score at baseline was 32.81 ± 4.87.

As summarized in Table 1, no statistically significant differences were observed between the treatment and control groups in baseline clinical parameters. The VAS score was 5.23 ± 0.90 in the combined treatment group and 5.09 ± 1.01 in the CsA monotherapy group (*p* = 0.566). NIBUT was 1.39 ± 0.84 s vs. 1.43 ± 0.82 s (*p* = 0.511), TMH was 0.09 ± 0.05 mm in both groups (*p* = 0.647), and DC density was 15.62 ± 5.07 vs. 15.95 ± 5.72 cells/mm^2^ (*p* = 0.560). These findings indicate good baseline comparability between the paired eyes.

**Table 1:** Baseline clinical characteristics of the studied eyes.

Clinical characteristics	Total participants (n = 21)	Combined treatment group (n = 21)	CsA monotherapy group (n = 21)	*p* value
Age (years; mean ± SD)	43.33 ± 15.33			
Gender				
Male, n (%)	8 (38.1%)			
Female, n (%)	13 (61.9%)			
Ocular Surface Disease Index	32.81 ± 4.87			
VAS score, 0–10 cm		5.23 ± 0.90	5.09 ± 1.01	0.566
Noninvasive Tear Break-Up Time (s)		1.39 ± 0.84	1.43 ± 0.82	0.511
Tear Meniscus Height (mm)		0.09 ± 0.05	0.09 ± 0.05	0.647
Corneal Dendritic Cell Density (cells/mm^2^)		15.62 ± 5.07	15.95 ± 5.72	0.560

SD, standard deviation; OSDI, ocular surface disease index; VAS, visual analog scale.

### 3.2 Comparison of clinical parameters before and after treatment

Significant improvements in multiple ocular surface parameters were observed in both groups after treatment. In the combined treatment group, NIBUT, TMH, and VAS scores increased significantly, whereas DC density decreased markedly (all *p* < 0.001). Similar improvements were also noted in the CsA monotherapy group (all *p* < 0.01 or p < 0.001).

**Figure 2:**
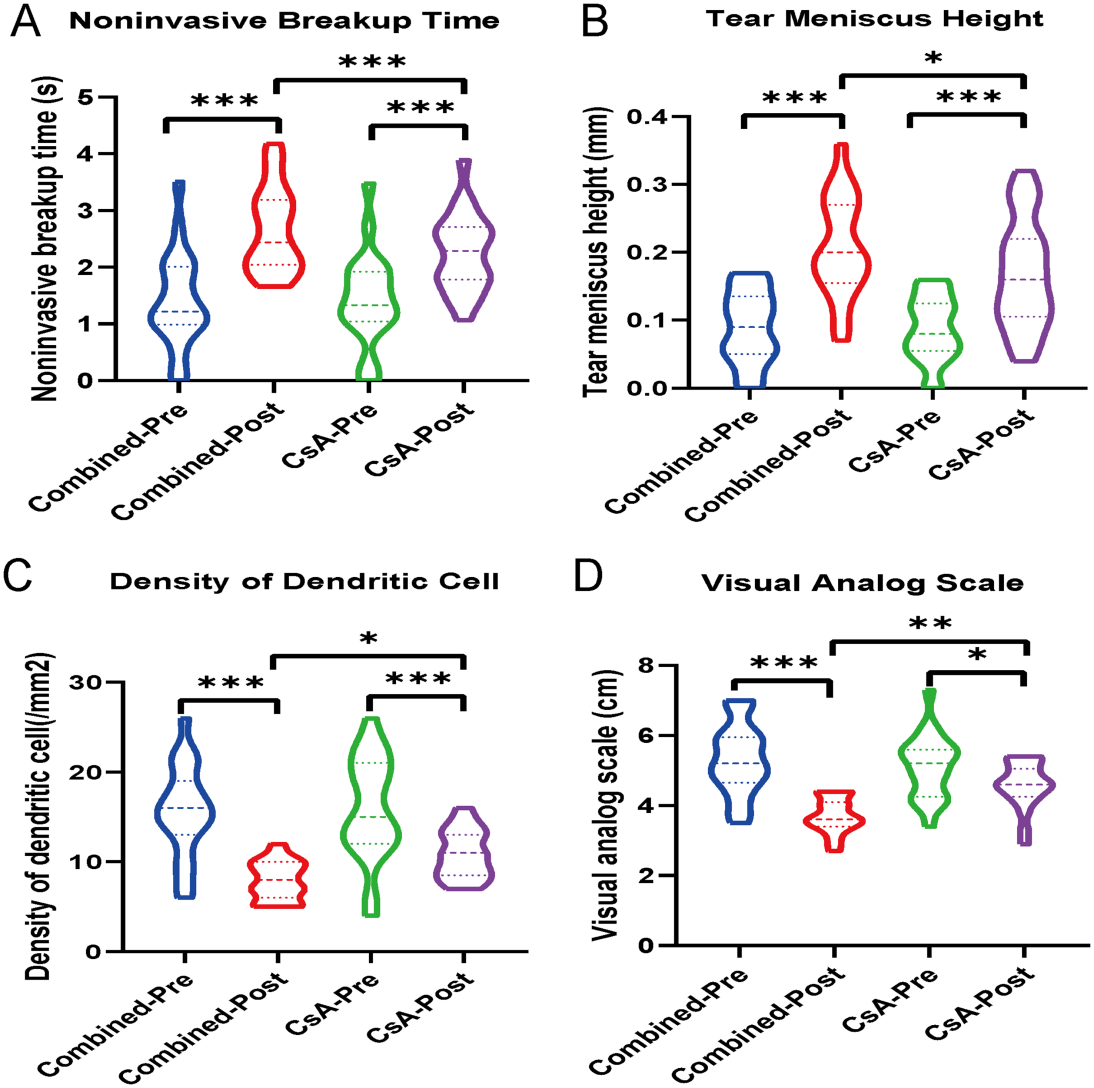
Violin plots showing the changes in **(A)** noninvasive tear breakup time (NIBUT), **(B)** tear meniscus height (TMH), **(C)** dendritic cell (DC) density, and **(D)** visual analog scale (VAS) scores in both the combined treatment group and the cyclosporine A (CsA) monotherapy group, before and after treatment. *p < 0.05, **p < 0.01, ***p < 0.001.

When comparing the magnitude of change between groups, the combined treatment group showed significantly greater improvements in NIBUT (*p* < 0.01), TMH (*p* < 0.05), and DC density (*p* < 0.01) than the CsA group. A more pronounced reduction in VAS scores was also observed in the combined treatment group (*p* < 0.01) (Figure 2).

### 3.3 Comparative analysis of clinical improvement and correlation between subjective parameters

**Figure 3:**
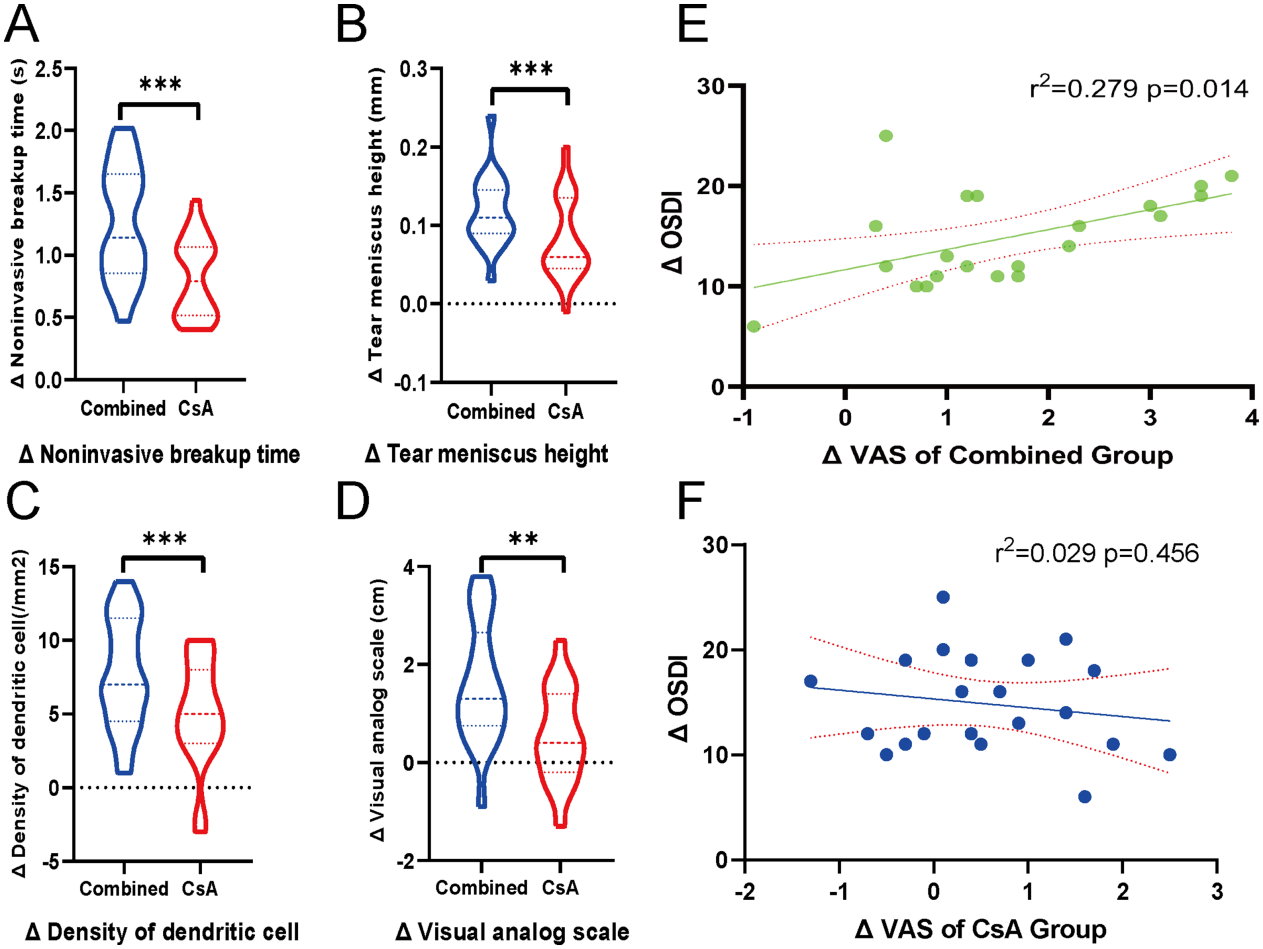
Comparison of ocular surface parameter improvements between groups and correlation between subjective scores. **(A–D)** Violin plots showing the inter-eye comparisons of changes (*Δ*, post–pre) in four clinical parameters: **(A)** Noninvasive tear breakup time (NIBUT), **(B)** Tear meniscus height (TMH), **(C)** Dendritic cell (DC) density, and **(D)** Visual analog scale (VAS) scores, comparing the combined treatment eyes and the contralateral cyclosporine A (CsA) monotherapy eyes. Each violin plot illustrates the distribution of Δ values, with dashed lines indicating the median and interquartile range. Statistical comparisons were performed using paired t-tests. ***p < 0.001, **p < 0.01. **(E,F)** Scatter plots showing the linear regression analysis between ΔVAS and ΔOSDI in each group. **(E)** Combined treatment group and **(F)** CsA monotherapy group. Solid lines indicate the regression fit, and dotted lines represent 95% confidence intervals. R^2^ and *p*-values are displayed in each panel.

Changes in clinical indicators (*Δ*, pre-post) were compared between the two treatment groups. The combined treatment group demonstrated significantly greater improvements than the CsA monotherapy group in ΔNIBUT (*p* < 0.001), ΔTMH (*p* < 0.001), ΔDC density (*p* < 0.001), and ΔVAS scores (*p* < 0.01), as shown in Figures 3A–D.

Pearson’s correlation analysis was performed to evaluate the association between the changes in subjective symptoms (ΔVAS) and overall symptom severity (ΔOSDI). A significant positive correlation was observed in the combined treatment group (r = 0.528, *p* = 0.014), whereas no significant correlation was found in the CsA monotherapy group (r = −0. 172, *p* = 0.456). Linear regression plots with 95% confidence intervals are shown in Figures 3E–F to illustrate these relationships.

## 4 Discussion

This paired-eye randomized controlled trial demonstrated that combination therapy with 0.05% CsA and an absorbable punctal plug led to significantly greater improvements in key ocular surface parameters than CsA monotherapy in patients with SS-DE. Specifically, the combined treatment group showed significantly greater improvements in ΔNIBUT (*p* < 0.001), ΔTMH (*p* < 0.001), and ΔDC density (*p* < 0.001), as well as a more substantial reduction in ΔVAS scores (*p* < 0.01). These findings suggest that punctal occlusion enhances the anti-inflammatory and tear-preserving effects of topical cyclosporine in SS-DE management.

To our knowledge, no prior clinical study has directly compared combination therapy using CsA and absorbable punctal plugs with CsA monotherapy in a paired-eye design, specifically for SS-DE. Most prior studies have evaluated these interventions separately, confirming the efficacy of either 0.05% CsA or punctal plugs in improving dry eye signs and symptoms (5, 11–13). However, a recent translational study by Feng et al. provided preliminary evidence supporting the synergistic anti-inflammatory effects of combining CsA with punctal occlusion. Their research, which included both clinical and animal experiments, demonstrated that the combination therapy more effectively improved tear production and tear film stability, while reducing pro-inflammatory cytokine levels in both human tears and rabbit ocular surface tissues, compared to either treatment alone (14). Building on this foundation, our study is the first to employ an intrasubject paired-eye clinical model in patients with SS-DE to assess the additive benefits of this combination. The paired-eye design minimized potential confounding variables, such as inter-individual differences in disease severity, immune status, and systemic treatment, thereby enhancing the internal validity of our findings (15, 16).

The improved ocular surface parameters observed in the combined treatment group may be attributed to the pharmacokinetic advantage of the prolonged ocular surface residence time of CsA, coupled with enhanced tear retention. The significant reduction in DC density in this group suggests that the anti-inflammatory effect of CsA was potentiated under conditions of enhanced tear film stability. This finding supports the hypothesis that suppressing inflammatory cell activity is critical for ocular surface restoration in SS-DE.

Notably, the correlation between improvements in ΔVAS and ΔOSDI scores was only significant in the combined treatment group, as shown by Pearson’s correlation and linear regression analyses. This suggests that combining CsA and punctal occlusion may result in a more consistent alignment between subjective symptoms and objective clinical improvement, potentially owing to a more effective suppression of underlying inflammation.

To minimize bias, this study employed a paired-eye design in which each participant served as their own control, thus eliminating interindividual variability such as systemic immune status, disease duration, and lifestyle factors. Additionally, observer masking was rigorously implemented—outcome assessors and imaging analysts were blinded to treatment allocation. Although full double-blinding was not feasible due to the nature of punctal plug insertion, subject masking was partially achieved by performing a sham procedure in the contralateral (monotherapy) eye, which involved identical preparation steps without actual plug placement. This approach helped reduce perception bias and maintained ethical standards, as both eyes received active cyclosporine A therapy and no participant was deprived of standard care. We selected a 3-month follow-up period to evaluate the therapeutic efficacy of the absorbable punctal plug, which remains effective for up to 6 months but undergoes gradual biodegradation over time. This duration ensures reliable assessment while the plug maintains sufficient functional integrity. Additionally, a 3-month window aligns with the follow-up period adopted in many previous studies of punctal occlusion and dry eye therapies, allowing for meaningful comparisons (17). Moreover, a longer follow-up would increase the risk of systemic treatment modifications in patients with Sjögren’s syndrome, potentially introducing confounding variables. Therefore, a 3-month duration was considered optimal for capturing short-term efficacy while minimizing systemic influences.

This study had a few limitations. The relatively small sample size limited our ability to include additional treatment arms, such as a punctal plug-only group or a sham control group. Therefore, the isolated effects of punctal occlusion could not be fully evaluated. Additionally, although the 3-month follow-up covered the main therapeutic window of the absorbable plug, long-term effects beyond plug absorption were not assessed. Lastly, inflammatory biomarkers in tear fluid (e.g., IL-6, MMP-9) were not measured, which limits mechanistic insights into how punctal occlusion may enhance the anti-inflammatory effects of cyclosporine A. Future studies incorporating cytokine profiling and longer-term follow-up will be necessary to fully understand the therapeutic synergy and durability of combined treatment.

In summary, our findings support the use of absorbable punctal plugs as an effective adjunct to CsA therapy in patients with SS-DE. This combined approach enhances both clinical outcomes and patient-reported symptoms and may represent an optimized therapeutic strategy for managing autoimmune-related dry eye disease. Long-term follow-up beyond the absorption period of the punctal plugs is warranted to assess the durability of therapeutic benefits.

## Data Availability

The original contributions presented in the study are included in the article/supplementary material, further inquiries can be directed to the corresponding author.

## Footnotes

1
www.sealedenvelope.com

## References

[1] Negrini S Emmi G Greco M Borro M Sardanelli F Murdaca G . Sjogren's syndrome: a systemic autoimmune disease. Clin Exp Med. (2022) 22:9–25. doi: 10.1007/s10238-021-00728-6, 34100160 PMC8863725

[2] Wu KY Serhan O Faucher A Tran SD. Advances in Sjogren's syndrome dry eye diagnostics: biomarkers and biomolecules beyond clinical symptoms. Biomolecules. (2024) 14:80. doi: 10.3390/biom14010080, 38254680 PMC10812982

[3] Zhang M Liang Y Wu H Zong R Zhang X He H . Ocular surface involvements in the development of Sjogren's syndrome-associated dry eye in the IL14alpha transgenic mouse. Invest Ophthalmol Vis Sci. (2025) 66:2. doi: 10.1167/iovs.66.3.2, 40029244 PMC11887930

[4] Doctor MB Kate A Tallapelly HG Basu S. The role of topical cyclosporine a in ocular surface inflammatory disorders. Semin Ophthalmol. (2025):1–12. doi: 10.1080/08820538.2025.2512759, 40468687

[5] Chen KY Chan HC Chan CM. How effective and safe are punctal plugs in treating dry eye disease? A systematic review and meta-analysis. Cont Lens Anterior Eye. (2025) 48:102438. doi: 10.1016/j.clae.2025.102438, 40393913

[6] Song JS Woo IH Eom Y Kim HM. Five misconceptions related to Punctal plugs in dry eye management. Cornea. (2018) 37:S58–61. doi: 10.1097/ICO.0000000000001734, 30211751

[7] Best AL Labetoulle M Legrand M M'Garrech M Barreau E Rousseau A. Punctal and canalicular plugs: indications, efficacy and safety. J Fr Ophtalmol. (2019) 42:e95–e104. doi: 10.1016/j.jfo.2018.12.003, 30692031

[8] Shiboski CH Shiboski SC Seror R Criswell LA Labetoulle M Lietman TM . American College of Rheumatology/European League Against Rheumatism classification criteria for primary Sjogren's syndrome: A consensus and data-driven methodology involving three international patient cohorts. Ann Rheum Dis. (2017) 76:9–16. doi: 10.1136/annrheumdis-2016-210571 27789466

[9] Cornea Group of Ophthalmology Branch of Chinese Medical, A.; Cornea Group of Chinese Ophthalmologist, A. Chinese expert consensus on the diagnosis and treatment of dry eye (2024). Zhonghua Yan Ke Za Zhi. (2024) 60:968–76. doi: 10.3760/cma.j.cn112142-20240517-00227, 39648024

[10] Faul F Erdfelder E Buchner A Lang AG. Statistical power analyses using G*power 3.1: tests for correlation and regression analyses. Behav Res Methods. (2009) 41:1149–60. doi: 10.3758/BRM.41.4.1149, 19897823

[11] Moawad P Shamma R Hassanein D Ragab G El Zawahry O. Evaluation of the effect of topical tacrolimus 0.03% versus cyclosporine 0.05% in the treatment of dry eye secondary to Sjogren syndrome. Eur J Ophthalmol. (2022) 32:673–9. doi: 10.1177/1120672121992680, 33530719

[12] Liu T Liu S Gan M He Y Fu H Xu M. Changes of dry eye parameters especially Meibomian gland functions after Punctal plugs insertion in aqueous-deficient dry eye patients. Front Med (Lausanne). (2022) 9:849700. doi: 10.3389/fmed.2022.849700, 35308530 PMC8925321

[13] Gao M Zhao L Liang R Zhu Q Zhao Q Kong X. Evaluation of the efficacy and safety of topical 0.05% cyclosporine eye drops (II) in the treatment of dry eye associated with primary Sjogren's syndrome. Ocul Immunol Inflamm. (2023) 31:1662–8. doi: 10.1080/09273948.2022.2094812, 35914303

[14] Feng C Wang W Gong L Lin T. Efficacy of topical cyclosporine combined with Punctal plugs in treating dry eye disease and inflammation. Curr Eye Res. (2025) 50:148–61. doi: 10.1080/02713683.2024.2411699, 39373208

[15] Chiang JCB Dremin V Semp DA Lam HY Ting PW Ayaz M . Low-level light therapy alone versus combination therapy with intense pulsed light in the treatment of dry eye disease with meibomian gland dysfunction: a randomised paired-eye and mechanism of action trial. Cont Lens Anterior Eye. (2025) 102456:102456. doi: 10.1016/j.clae.2025.102456, 40494722

[16] Assayag E Abulafia A Teren D Gelman E Givoni H Zadok D. Efficacy of 1% povidone-iodine in the treatment of anterior blepharitis-randomized single-center controlled trial. J Clin Med. (2024) 13:13 (23). doi: 10.3390/jcm13237227, 39685686 PMC11642136

[17] Ervin AM Law A Pucker AD. Punctal occlusion for dry eye syndrome. Cochrane Database Syst Rev. (2017) 2017:CD006775. doi: 10.1002/14651858.CD006775.pub3, 28649802 PMC5568656

